# THE IMPACT OF EHEALTH ON SELF-MANAGEMENT AND HEALTH OUTCOMES OF PATIENTS WITH TYPE 2 DIABETES: A SYSTEMATIC REVIEW

**DOI:** 10.1093/geroni/igae098.3025

**Published:** 2024-12-31

**Authors:** Yu-Shan Cheng, Heather Cuevas

**Affiliations:** The University of Texas Austin, Austin, Texas, United States; The University of Texas Austin, Austin, Texas, United States

## Abstract

Self-management is crucial to maintain glycemic control in type 2 diabetes (T2DM). Prior studies have shown that various electronic health (eHealth) technologies promote diabetes self-management (DMSM) and continuity of care. However, previous reviews focused mainly on telehealth, a single aspect, and were published more than ten years ago. Therefore, the purpose of this study was to summarize the effect of eHealth interventions on improving aspects of DMSM and health outcomes, such as depression and A1C, for adults over 60 years of age with T2DM. A systematic review was conducted on papers published from October 2018 to October 2023. Three electronic databases were used. 2020 Preferred Reporting Items for Systematic Reviews and Meta-Analyses (PRISMA) served as guides. Twenty randomized control trials in diverse ethnic communities (n=3978) across 20 countries were included in the narrative synthesis. Seven health technologies were employed to provide DMSM across studies, including mobile health applications, text messaging, phone calls, online conferences, video, wearable devices, and virtual worlds. Overall, improvement in DMSM and health outcomes was mixed. In terms of depression, only three studies showed significant improvement, while four studies did not exhibit significant improvement in A1C. Additionally, positive outcomes were not maintained after seven months (the furthest post-intervention measurement). These findings differed according to eHealth literacy, economic burden, willingness, and social support. Further work is needed to consider other variables to address both short- and long-term effects for the patients while implementing healthcare technology.

